# Sleep-disordered breathing in patients with newly diagnosed lung cancer

**DOI:** 10.1186/s12890-018-0645-1

**Published:** 2018-05-16

**Authors:** Michael Dreher, Stefan Krüger, Susanne Schulze-Olden, András Keszei, Jan Hendrik Storre, Holger Woehrle, Michael Arzt, Tobias Müller

**Affiliations:** 1 Division of Pneumology, University Hospital RWTH Aachen, Aachen, Germany; 2 Department of Pneumology, Florence-Nightingale-Hospital, Düsseldorf, Germany; 3 Department of Medical Informatics, University Hospital RWTH Aachen, Aachen, Germany; 4 Department of Intensive Care, Sleep Medicine and Mechanical Ventilation, Asklepios Fachkliniken Munich-Gauting, Gauting, Germany; 5 Department of Pneumology, University Medical Hospital, Freiburg, Germany; 6 Sleep and Ventilation Center Blaubeuren, Lung Center Ulm, Ulm, Germany; 7 Center of Sleep Medicine, Department of Internal Medicine II, University Hospital Regensburg, Regensburg, Germany

**Keywords:** Lung neoplasm, sleep-related disorders, sleep-related hypoxaemia, Lung cancer, Sleep apnea, Sleep disorders

## Abstract

**Background:**

There are currently no data on the prevalence of sleep-disordered breathing (SDB) in patients with newly-diagnosed lung cancer. This might be of interest given that SDB is associated with increased cancer incidence and mortality. Furthermore, intermittent hypoxia has been linked with tumor growth and progression. The aim of the current study was to investigate the prevalence of SDB in patients with newly-diagnosed lung cancer.

**Methods:**

Patients with newly-diagnosed lung cancer from three centers in Germany were screened for SDB using a two-channel screening system (ApneaLink™). SDB was defined as an apnea-hypopnea index of > 5/h, and was classified as mild if the AHI was 5–15/h whereas an AHI ≥15/h was classified as severe SDB. The presence of SDB-related symptoms was assessed using the Epworth Sleepiness Scale (ESS) and the Pittsburgh Sleep Quality Index (PSQI).

**Results:**

A total of 100 patients were included. The overall prevalence of SDB was 49%; 32 patients (32%) had mild SDB with a median AHI of 7.7/h (quartile [Q1 5.4/h, Q3 10.4/h]) and a median oxygen desaturation index of 8.5 [Q1 4.2/h; Q3 13.4/h] and seventeen patients (17%) had moderate to severe SDB with a median AHI of 25.2 [Q1 18/h, Q3 45.5/h] and a median oxygen desaturation index of 20.6/h [Q1 9.6/h, Q3 36.6/h]. Patients with moderate to severe SDB had mild daytime sleepiness (ESS score 8.24 ± 3.96 vs. 5.74 ± 3.53 in those without SDB vs. 6.22 ± 2.72 in those with mild SDB; *p* = 0.0343). The PSQI did not differ significantly between the three groups (*p* = 0.1137).

**Conclusions:**

This study showed a high prevalence of SDB in patients with newly-diagnosed lung cancer. In these patients SDB was associated with intermittent hypoxia and increased daytime sleepiness. Additional research is needed to determine whether SDB influences prognosis and morbidity in patients with lung cancer.

**Trial registration:**

NCT02270853 (ClinicalTrials.gov), date of registration: 14th October 2014.

## Background

Sleep-disordered breathing (SDB) is a common public health problem with a prevalence of 5–20% in the general population, [1, 2] although many patients remain undiagnosed. Obstructive sleep apnea is the most common form of SDB, and has been linked to a number of adverse clinical outcomes including increased cardiovascular and all-cause mortality [3].

The association between SDB and cancer remains to be clearly defined, although previous studies have shown SDB to be associated with increased cancer incidence and cancer mortality. [4–7] Furthermore, it has been shown that sleep time with oxygen saturation below 90% has the strongest association with cancer mortality, highlighting the importance of hypoxemia. [4] This is supported by animal studies showing that intermittent hypoxia, as occurs in patients with obstructive sleep apnea, was associated with tumor growth and progression. [8] Underlying mechanisms include angiogenesis, epithelial-mesenchymal transition, migration/invasion, maintenance of cancer stem cells, metastasis, and immune surveillance, as well as resistance to chemotherapy and radiotherapy. As a result, hypoxemia has been linked with poor prognosis in different malignancies [9, 10].

To the best of our knowledge, there is currently no published data on the prevalence of undiagnosed SDB in patients with newly-diagnosed lung cancer. This is relevant because the presence of SDB might influence morbidity and mortality in lung cancer patients and the treatment of intermittent hypoxia could therefore influence the outcome or response to therapy. This study investigated the prevalence of SDB in patients with newly-diagnosed lung cancer.

## Methods

The study received approval from the Institutional Review Board for Human Studies at the University Hospital RWTH Aachen, Germany (EK 312/13). Written informed consent was provided by each subject before participation. All procedures conformed to the standards set by the Declaration of Helsinki in its latest revision.

### Patients

From 5th August 2014 until 24th February 2017 consecutive patients with newly-diagnosed lung cancer from three different study centres (University Hospital RWTH - Aachen, Florence-Nightingale-Hospital - Düsseldorf, Cologne-Merheim Hospital - Cologne) were included. Inclusion into the study was done before any treatment for lung cancer (surgery, radiotherapy, or chemotherapy) was initiated. Also excluded were patients with a history of any malignancy or previously diagnosed sleep apnea.

### Study design

Patient demographic data, smoking history, and co-morbidities were assessed. Screening for SDB was performed during hospital stay or at home. Daytime sleepiness was assessed using the Epworth Sleepiness Scale (ESS) and Sleep Quality was assessed using the Pittsburgh Sleep Quality Index (PSQI) [11, 12].

### Assessing sleep-disordered breathing

SDB screening was performed using a two-channel screening system (ApneaLink Oxi™; ResMed Ltd., Sydney, Australia) which has been validated previously. [13, 14] The ApneaLink™ measures nasal flow via a cannula and records pulse oximetry; further details are described elsewhere. [14] Default device settings were used to define apnea, hypopnea and desaturation as follows: apnea was defined as a decrease of at least 80% in airflow for ≥10 s; hypopnea was defined as a decrease in airflow by 50–80% for ≥10 s; desaturation was defined as a ≥ 4% decrease in oxygen saturation. Mild SDB was defined as AHI of 5 to < 15/h and moderate to severe SDB as an AHI of ≥15/h. [15] The screening device was read out by one investigator at the University Hospital Aachen who was blinded to the patients’ status.

### Statistical analysis

Continuous data are presented as mean ± standard deviation and categorical data are tabulated as frequencies and percentages. Groups of patients defined based on cutoff values of AHI were compared using ANOVA, Kruskal-Wallis rank sum test and Chi-square test. Nominal *p*-values are presented for each comparison. Linear models were built using the logarithmic transformed AHI. The following variables were used as independent variables in the models: age (linear effect), sex and body mass index (BMI; linear) were included in all models, and Union for International Cancer Control (UICC) tumor stage (4 categories), arterial hypertension (yes/no), chronic obstructive pulmonary disease (COPD; yes/no) and coronary artery disease (yes/no) were added to the models using all subsets. We present results for the model with the highest adjusted r squared. In additional analyses, AHI was modeled using a generalized linear model with Poisson family and logarithmic link function (data not shown). The prevalence of SDB was estimated from the sample proportions and exact binomial 95% confidence interval (CI) values were calculated.

## Results

### Patient characteristics

During the study period 625 patients were newly diagnosed with lung cancer at the three study centers. Data from 100 patients who fulfilled the inclusion criteria and consented to take part in the study were included. Patient demographic data are shown in Table 1. The most common form of lung cancer was adenocarcinoma, followed by squamous cell carcinoma and small cell lung cancer (Table 1). Sleep and respiratory parameters for the total population are presented in Table 2.

**Table 1:** Patient demographic data

	Patients (*n* = 100)
Age, years	68.1 ± 8.6
Male, *n*	67
BMI, kg/m^2^	26.0 ± 4.6
Histology, *n*	
Small cell lung cancer	23
Non-small cell lung cancer	77
- Adenocarcinoma	45
- Squamous cell carcinoma	24
- Other	8
Tumor stage (UICC), *n*	
I	9
II	13
III	28
IV	50
Smoking history, *n*	
Current smoker	48
Ex-smoker	36
Never smoked	16
Comorbidities, *n*	
Hypertension	56
Diabetes	14
Coronary artery disease	22
Chronic heart failure	8
COPD	29

Values are mean ± standard deviation, or number of patients

*BMI* body mass index, *COPD* chronic obstructive pulmonary disease, *UICC* Union for International Cancer Control

**Table 2:** Sleep-disordered breathing analysis, daytime sleepiness and sleep quality

	Patients (*n* = 100)
ESS score	6.3 ± 3.5
PSQI score	7.0 ± 3.2
AHI, /h	4.9 [2.0, 10.4]
ODI, /h	5.3 [3.0, 10.1]
Mean SpO_2_, %	91.3 ± 2.4
Lowest SpO_2_, %	78.2 ± 8.2
Time with SpO_2_ < 90%, min	34.5 [9.8, 78.0]
Heart rate, beats/min	72.7 ± 11.8
Respiration rate, breaths/min	17.7 ± 4.9
Snoring, /h	66.6 [22.7, 130.3]

Values are mean ± standard deviation, or median [first quartile, third quartile]

*AHI* apnea-hypopnea index, *ESS* Epworth Sleepiness Scale, *ODI* oxygen desaturation index, *PSQI* Pittsburgh Sleep Quality Index, *SpO*_*2*_ oxygen saturation

### SDB prevalence

The overall prevalence of SDB (AHI > 5/h) in the study population was 49% (95% CI 38.9–59.2%) and was higher in men (55%) versus women (36%). The median sleep time with oxygen saturation below 90% was 33.0 min for patients with SDB. Moderate to severe SDB was present in 17% (95% CI 10.2–25.8%), whereas mild SDB (AHI 5 to < 15/h) was present in 32% (95% CI 23.0–42.1%) of the patients. There were several statistically significant differences between patients who had mild or moderate to severe SDB and those who did not, including AHI, and ODI (Table 3). Patients with moderate to severe SDB showed mild daytime sleepiness (ESS score 8.24 ± 3.96), and were more sleepy than those without SDB or mild SDB (Table 3). Sleep quality (PSQI), BMI, tumor histology and tumor stage were not significantly different between the three groups (Table 3). The prevalence of distinct comorbidities was similar in the three groups. However, there was a trend towards a higher prevalence of COPD in patients without or with mild SDB compared to patients with moderate to severe SDB (29.4 and 37.5% vs. 11.8%) (Table 3).

**Table 3:** Comparison of patients without (AHI < 5/h), with mild (AHI 5–15/h)or moderate to severe (AHI > 15/h) sleep-disordered breathing

	AHI < 5/h	AHI 5–15/h	AHI > 15/h	*p*-value
n	51	32	17	N/A
Age, years	66.31 ± 8.48	69.16 ± 8.06	71.35 ± 8.775	0.0736
Male, *n* (%)	30 (58.8)	24 (75.0)	13 (76.5)	0.2062
BMI, kg/m^2^	25.53 ± 4.45	26,67 ± 4,95	26.41 ± 4.14	0.5133
AHI, /h	2 [1, 3]	7.7 [5.4, 10.4]	25.2 [18, 45.5]	< 0.0001
ODI, /h	3.1 [1.5, 5.1]	8.5 [4.2, 13.4]	20.6 [9.6, 36.6]	< 0.0001
Mean SpO_2_, %	91.47 ± 2.56	91.09 ± 2.36	91.35 ± 2.14	0.7900
Time with SpO_2_ < 90%, min	36 [8, 84]	25.5 [8.3, 92.3]	41 [10.5,63]	0.9999
ESS score	5.74 ± 3.53	6.22 ± 2.72	8.24 ± 3.96	0.0343
PSQI score	6.49 ± 2.69	7.97 ± 3.97	6.71 ± 2.62	0.1137
Snoring, /h	69 [19.6, 148]	61.2 [26.8, 156.4]	69 [30.5,89.7]	0.9343
Histology, *n* (%)				
SCLC	14 (27.5)	4 (12.5)	5 (29.4)	0.2280
NSCLC	37 (72.5)	28 (87.5)	12 (70.6)	
- Adenocarcinoma	22 (43.1)	18 (56.3)	5 (29.4)	0.7034
- Squamous cell carcinoma	12 (23.5)	7 (21.9)	5 (29.4)	
- Other	3 (5.9)	3 (9.4)	2 (11.8)	
Tumor stage (UICC), *n* (%)				
I	3 (5.9)	4 (12.5)	2 (11.8)	0.1556
II	5 (9.8)	3 (9.4)	5 (29.4)	
III	19 (37.3)	7 (21.9)	2 (11.8)	
IV	24 (47.1)	18 (56.3)	8 (47.1)	
Smoking history, *n* (%)				
Current smoker	27 (52.9)	14 (43.8)	7 (41.2)	0.8782
Ex-smoker	16 (31.4)	13 (40.6)	7 (41.2)	
Never smoked	8 (15.7)	5 (15.6)	3 (17.6)	
Comorbidities, *n* (%)				
Hypertension	26 (50.1)	21 (65.6)	9 (52.9)	0.4088
Diabetes	4 (7.8)	6 (18.8)	4 (23.5)	0.1749
Coronary artery disease	9 (17.6)	7 (21.9)	6 (35.3)	0.3144
Chronic heart failure	4 (7.8)	3 (9.4)	1 (5.9)	0.9105
COPD	15 (29.4)	12 (37.5)	2 (11.8)	0.1670
Pulmonary function				
FEV1 (% predicted)	64.69 ± 17.88	67.51 ± 18.65	77.09 ± 20.02	0.0926
PaO_2_ (mmHg)	67.62 ± 12.44	66.69 ± 9.01	69.34 ± 17.69	0.9264

Values are mean ± standard deviation, median [first quartile, third quartile], or number of patients (%)

*AHI* apnea-hypopnea index, *BMI* body mass index, *COPD* chronic obstructive pulmonary disease, *ESS* Epworth Sleepiness Scale, *FEV1* Forced Expiratory Volume in 1 s, *NSCLC* non-small cell lung cancer, *ODI* oxygen desaturation index, *PaO*_*2*_ capillary partial pressure of oxygen, *PSQI* Pittsburgh Sleep Quality Index, *SpO*_*2*_ oxygen saturation, *SCLC* small cell lung cancer, *UICC* Union for International Cancer Control

**Fig. 1:**
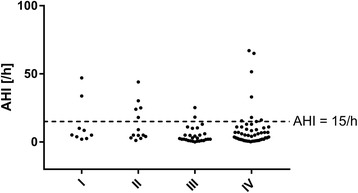
Relationship between the apnea-hypopnea index (AHI) and UICC tumor stage

**Fig. 2:**
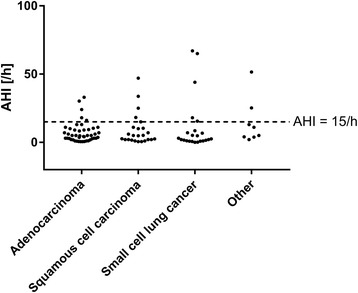
Relationship between the apnea-hypopnea index (AHI) and different subtypes of lung cancer

**Fig. 3:**
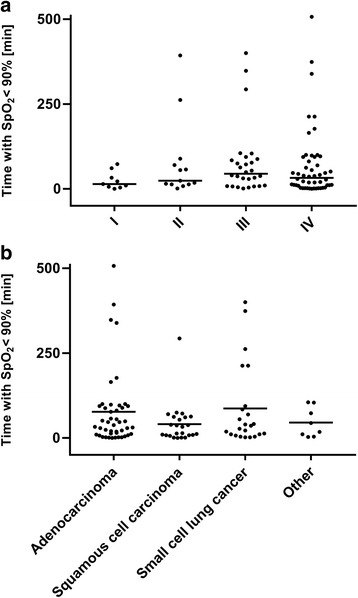
Relationship between sleep time with oxygen saturation < 90% (*T* < 90%) and different tumor stages (**a**) or tumor histology (**b**)

Comparing AHI across different tumor stages showed a lower AHI in stage III compared with earlier stages (median 5.1/h, 5.0/h, 2.3/h, and 5.1/h for stages I, II, III, and IV, respectively; *p* = 0.04) (Fig. 1). There were no significant differences in AHI by histologic tumor type (Fig. 2). Median sleep time spent with oxygen saturation below 90% was 14, 24, 45, and 33 min in stages I, II, III, and IV, respectively (Fig. 3a), and time with oxygen saturation below 90% also did not differ between different histological tumor types (Fig. 3b).

### Clinical predictors for SDB

The best fitting regression model for the prediction of SDB included age, sex, BMI, and UICC categories II, III, and IV, with estimated regression coefficients of 0.03 (95% CI –0.01, 0.06), 0.57 (− 0.03, 1.17), 0.02 (− 0.05, 0.08), 0.14 (− 1.05,1.34), − 1.03 (− 2.09, 0.04), and − 0.29 (− 1.30, 0.73), respectively. The adjusted r-squared was 0.11, indicating that the model had weak predictive value.

## Discussion

This study reports the prevalence of SDB in patients with newly-diagnosed lung cancer for the first time and showed that nearly half of all patients had any SDB (AHI > 5/h) and that the prevalence of moderate to severe SDB in this population was 17%.

The reported prevalence of SDB is dependent on the AHI cutoff value used. [13, 14] The 17% prevalence rate for moderate to severe SDB in our cohort with newly-diagnosed lung cancer lies within the estimated prevalence range in the general population (5 to 20%). [1, 2] When an AHI cutoff value of 5/h was used, the estimated prevalence of SDB in patients with newly-diagnosed lung cancer rose markedly to 49%. Of note the average BMI was in the normal range and not significantly different between the groups. Hence one could speculate that the prevalence of SDB is even increased in lung cancer patients. However, as we do not have a control group with similar anthropometric characteristics not suffering from lung cancer this cannot be deducted from the results of the current study.

The exact prevalence of SDB in patients with lung cancer may be less important than the fact that our study shows that a high proportion of patients with newly-diagnosed lung cancer have undiagnosed SDB. This might be particularly relevant given that previous studies have shown that SDB is associated with both increased cancer incidence and increased cancer mortality. [4–7] If this is also the case for patients with lung cancer, screening for SDB in these patients is likely to be clinically important.

Both increased cancer incidence and increased cancer mortality have been shown to be dependent on the severity of hypoxemia occurring during SDB,^4,6^ and animal data demonstrated an association between intermittent hypoxia and tumor growth and progression. [8] The mean ODI in the present study was 9 ± 12/h overall and 25 ± 20/h in patients with moderate to severe SDB. Although intermittent hypoxia was present in our cohort we did not find any correlation between the severity of SDB and cancer type or tumor stage. Furthermore, sleep time with oxygen saturation below 90% was not correlated with cancer type or tumor stage. However, this was not the goal of the current study and the trial was not powered for these analyses. Nevertheless, our results might stimulate research to assess clinical outcomes in lung cancer patients with underlying SDB. Continuous positive airway pressure is the standard of care for patients with obstructive sleep apnea, improving both the AHI and ODI. [16] Therefore, studies investigating the impact of treating intermittent hypoxia in patients with lung cancer on the clinical course of the disease would be of high clinical interest.

Sleep apnea is associated with impaired health-related quality of life due to symptoms such as reduced sleep quality, daytime sleepiness, and fatigue. [16] Chronic fatigue is a common problem in patients with lung cancer, [17] which might be aggravated in the presence of SDB or, in some instances, may even be caused by SDB. Although sleep quality measured using the PSQI was not different in patients with and without SDB, daytime sleepiness based on the ESS score, was significantly higher in newly-diagnosed lung cancer patients who also had SDB. Therefore, treating SDB in patients with lung cancer might have the potential to improve SDB symptoms and thus also health-related quality of life. However, this remains speculative and should be evaluated in a prospective clinical trial.

In our cohort of patients with newly-diagnosed lung cancer, age, BMI, sex, and tumor stage did not predict SDB, but this may be due to a lack of statistical power for the analyses.

Our study has several limitations which have to be addressed: Firstly, the device used to screen for SDB has limited diagnostic abilities, e. g. it does not allow to distinguish obstructive from central sleep apnea. Secondly, the proportion of screening failures was high, and we did not collect data about the prevalence of SDB in a matched control group without lung cancer. Thirdly, SDB was assessed both at home and in hospital which might have yielded different results.

## Conclusions

In conclusion, the rate of moderate to severe SDB in patients with newly-diagnosed lung cancer was 17% and nearly half of all patients had any SDB. Moderate to severe SDB in this cohort was associated with intermittent hypoxia and increased daytime sleepiness. Since previous studies have linked pathophysiological features of SDB, such as intermittent hypoxia, with tumor growth and progression, further research is warranted to determine whether SDB influences the outcome of patients with lung cancer and whether treatment of SDB can improve health-related quality of life, slow disease progression, or even increase survival in lung cancer patients.

## Abbreviations

AHI: Apnea hypopnea index
ESS: Epworth sleepiness scale
ODI: Oxygen desaturation index
SDB: Sleep disordered breathing
